# New Insights into the Microvascular Mechanisms of Drag Reducing Polymers: Effect on the Cell-Free Layer

**DOI:** 10.1371/journal.pone.0077252

**Published:** 2013-10-04

**Authors:** Judith Brands, Dustin Kliner, Herbert H. Lipowsky, Marina V. Kameneva, Flordeliza S. Villanueva, John J. Pacella

**Affiliations:** 1 Department of Medicine, Cardiovascular Institute, University of Pittsburgh School of Medicine, University of Pittsburgh, Pittsburgh, Pennsylvania, United States of America; 2 Department of Bioengineering, Penn State University, University Park, Pennsylvania, United States of America; 3 McGowan Institute for Regenerative Medicine, University of Pittsburgh, Pittsburgh, Pennsylvania, United States of America; 4 Department of Bioengineering, University of Pittsburgh, Pittsburgh, Pennsylvania, United States of America; 5 Department of Surgery, University of Pittsburgh, Pittsburgh, Pennsylvania, United States of America; University of Arizona, United States of America

## Abstract

Drag-reducing polymers (DRPs) significantly increase blood flow, tissue perfusion, and tissue oxygenation in various animal models. In rectangular channel microfluidic systems, DRPs were found to significantly reduce the near-wall cell-free layer (CFL) as well as modify traffic of red blood cells (RBC) into microchannel branches. In the current study we further investigated the mechanism by which DRP enhances microvascular perfusion. We studied the effect of various concentrations of DRP on RBC distribution in more relevant round microchannels and the effect of DRP on CFL in the rat cremaster muscle *in vivo*. In round microchannels hematocrit was measured in parent and daughter branch at baseline and after addition of DRP. At DRP concentrations of 5 and 10 ppm, the plasma skimming effect in the daughter branch was eliminated, as parent and daughter branch hematocrit were equivalent, compared to a significantly lowered hematocrit in the daughter branch without DRPs. In anesthetized rats (N=11) CFL was measured in the cremaster muscle tissue in arterioles with a diameter of 32.6 ± 1.7 µm. In the control group (saline, N=6) there was a significant increase in CFL in time compared to corresponding baseline. Addition of DRP at 1 ppm (N=5) reduced CFL significantly compared to corresponding baseline and the control group. After DRP administration the CFL reduced to about 85% of baseline at 5, 15, 25 and 35 minutes after DRP infusion was complete. These *in vivo* and *in vitro* findings demonstrate that DRPs induce a reduction in CFL width and plasma skimming in the microvasculature. This may lead to an increase of RBC flux into the capillary bed, and thus explain previous observations of a DRP mediated enhancement of capillary perfusion.

## Introduction

In a canine model of severe coronary artery stenosis, we have shown that nanomolar concentrations of blood-soluble drag-reducing polymers (DRPs) restored myocardial perfusion to normal, measured using radiolabeled microspheres and myocardial contrast echocardiography (MCE) [1]. This observation aligns with previous work demonstrating beneficial (hemodynamic) effects of DRPs in multiple animal studies [2-8]. The mechanism by which DRP enhances microvascular perfusion is, however, incompletely understood.

DRP are high molecular weight polymers that have a primarily linear structure with few or no branches, and are known to reduce hydrodynamic resistance in turbulent flow, a phenomenon known as the Toms effect [9]. Blood flow in the microcirculation is, however, not turbulent, therefore indicating a different mechanism of DRP induced flow enhancement. Based on *in vitro* observations in a model of a vessel bifurcation, an alternate mechanism was suggested. That is, DRPs diminish flow separations and recirculation zones at vessel bifurcations [10]. More recently, a new DRP induced microrheological phenomenon in blood has been discovered that might explain the mechanism of DRP induced enhancement of microvascular perfusion; namely, minute concentration of DRP in red blood cell (RBC) suspensions flowing in a straight microchannel significantly reduces the thickness of the near wall cell-free plasma layer [8]. This plasma layer was discovered over 80 years ago by Fåhraeus [11] in both *in vivo* (microvessels) and *in vitro* (capillary tubes). To shed light on the microvascular mechanisms of DRP, Marhefka et al. [12] studied DRP additives to blood or RBC suspensions *in vitro* by observing the traffic of RBC in microfluidic systems. During the flow of RBC suspensions in straight rectangular microchannels with bifurcations and expansions, they observed a redistribution of RBCs and a reduction in both plasma layer width and plasma skimming (a reduced hematocrit in a small side branch of the main vessel [13,14]) after DRP was added to the flow loop. Based on this finding, it was suggested that the reduction in plasma skimming *in vivo* would significantly increase traffic of RBCs into vessel branches and ultimately to capillaries, thus increasing microvascular perfusion. This hypothesis was supported by the findings from our canine model of severe LAD coronary artery stenosis, namely that DRP was associated with an increase in MCE derived capillary blood volume after DRP administration in our canine model [1].

In the current study, we performed *in vitro* measurements in round bifurcating microchannels using several concentrations of DRP. Our goal was to determine whether the observed redistribution of RBCs occurs in a more physiologically relevant setup using round channels, instead of rectangular channels as reported previously [12]. Furthermore, and most importantly, we studied whether a reduction in the plasma layer as was seen *in vitro* occurs *in vivo*. To determine the effect of DRP *in vivo* the plasma layer, or cell-free layer (CFL), was measured in microvessels of the rat cremaster tissue before and after the infusion of DRP. We found, using round microchannels, a reduction in plasma skimming with DRP. In addition, we observed that DRP causes a reduction in CFL in an *in vivo* animal model of the rat cremaster muscle.

## Materials and Methods

### Drag reducing polymers (DRP)

DRP solution was comprised of polyethylene oxide (PEO, Sigma-Aldrich, Saint Louis, MO), with an average molecular weight (MW) of 4000 kDa. PEO was dissolved in saline at a concentration of 0.1% and then dialyzed against normal saline for 24 hours using a membrane with 50 kDa MW cutoff (Regenerated Cellulose Dialysis Membrane, Spectra/Por, Spectrum Laboratories Inc.). Viscosity of the PEO solution was measured by a rotational viscometer (Wells-Brookfield Cone/Plate viscometer, DVIII+, Middleboro, MA) to confirm complete dissolution and rule out mechanical degradation of the polymer during preparation. The stock PEO solution of 1000 ppm concentration was diluted to a concentration of 50 ppm with saline and mixed for 1–2 hours on a slow rocker prior to use.

### Microchannel bifurcation study

#### Bovine blood

Whole bovine blood was obtained from a local slaughterhouse (Thoma Meat Market, Saxonburg, PA) with their permission to be used for research. Heparin (10 U/mL, APP pharmaceuticals, Schaumburg, IL) was added to maintain anticoagulation. Blood was washed as described by Johnson et al. [15]. In short, to retard bacterial growth Gentamicin (250 mg/L, Sigma, St. Louis, MO) was added, and blood was filtered through a 40 µm filter to remove gross particles. Isolating RBCs was achieved by washing the whole blood in KH solution (without calcium) using a cell-saver machine (Haemonetics Corp., Braintree, MA). Isolated RBCs were then diluted with KH solution to obtain a hematocrit of 28.5 ± 0.9%. Calcium chloride (1.8 mM) was added after an additional heparin bolus (10 U/mL) was given. Albumin (0.3%, Sigma, St. Louis, MO) was used to maintain osmolarity, and a pH of 7.46 ± 0.02 was obtained with the addition of sodium bicarbonate (3%) to the final suspension.

#### Experimental protocol

**Figure 1:**
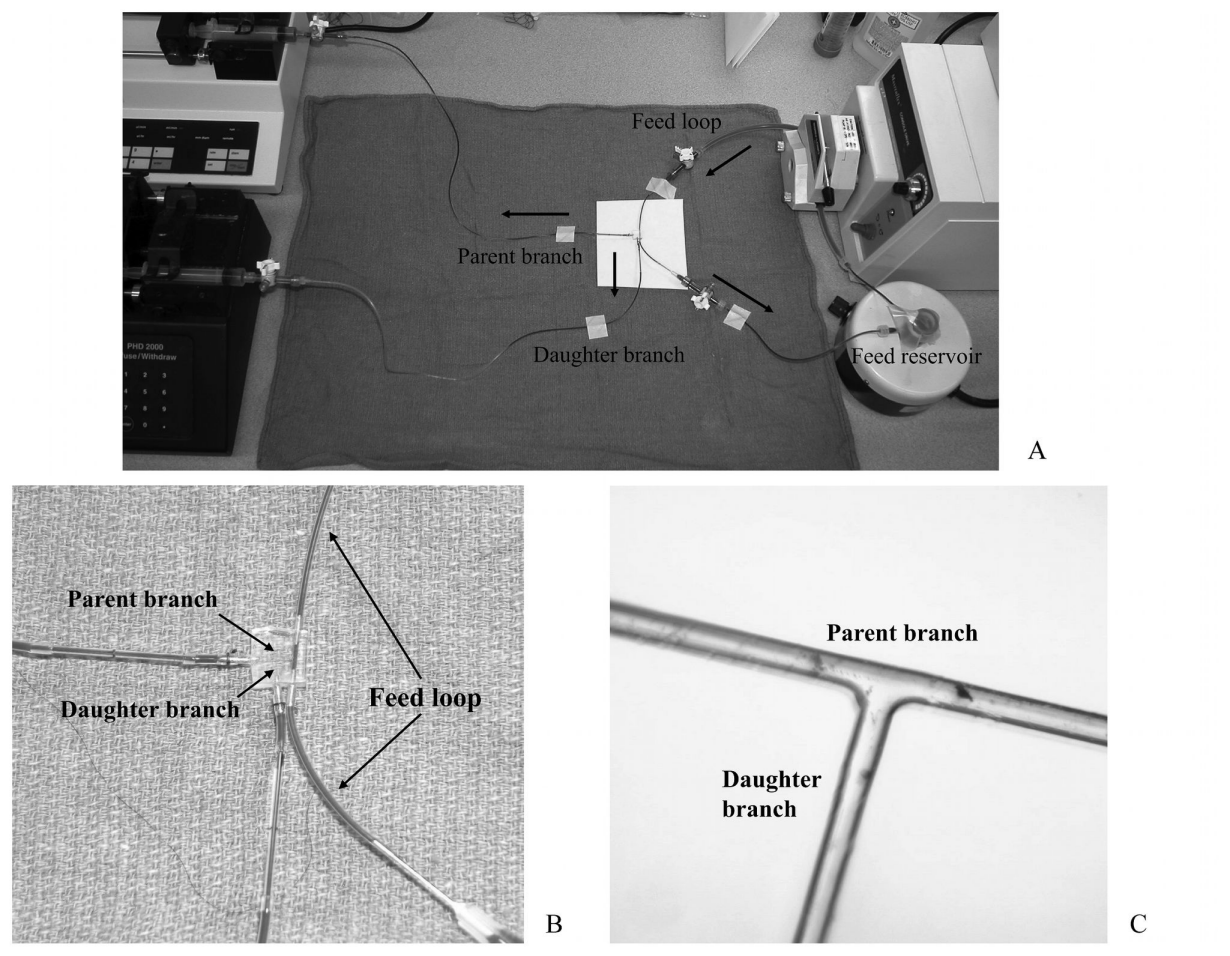
Microchannel bifurcation setup A. Microchannel experimental set-up. The feed reservoir and loop are positioned on the right. Blood enters from the top and as it traverses through the feed channel, a fraction is aspirated into a 50 µm parent branch which passes straight through the plastic mold to the discharge port which is connected the top syringe pump. The parent branch gives rise to a 50 µm daughter branch which is connected to the syringe pump on the bottom. Black arrows indicate the direction of blood flow. B. A microscopic view (10x magnification) of 50 µm bifurcation showing smooth, continuous contours. C. Photo of microchannel showing feed loop, parent branch, and daughter branch.

Experiments were carried out using round microchannels with a diameter of 50 µm constructed using a suspended-wire technique [16] (see Figure 1). The microchannels were connected through a feed loop to a flow pump (Masterflex peristaltic pump, Cole-Parmer, Court Vernon Hills, IL). A small flow reservoir was filled with approximately 10 ml of prepared RBC suspension and stirred constantly to prevent sedimentation of RBCs. Polyethylene tubing (PE-10, Becton Dickinson, Franklin Lakes, NJ) was used to connect the feed reservoir to the microchannel. The suspended RBCs were withdrawn from the feed reservoir and passed through the flow pump into the feed loop portion of the microchannel. The opposite end of the feed loop portion of the microchannel was again connected back to the feed reservoir using the same type polyethylene tubing, thus completing the loop. The parent and daughter branches were directly connected to capillary tubes using polyethylene tubing (PE-10). The opposite ends of the capillary tubes were connected to two separate syringe pumps with attached withdrawal switches (Harvard apparatus, Holliston, Ma). The withdrawal flow rates were set to 0.04 and 0.01 mL/min, for the parent and daughter branches respectively (see Figure 1).

The flow pump was started, filling the feed loop with the RBC suspension, after which the withdrawal pumps were started. The system was allowed to run initially until both the polyethylene tubing between the microchannel and capillary tube, and the capillary tube itself, were filled. After the capillary tubes in each branch were full, the withdrawal pumps were temporarily stopped, the capillary tubes removed, and one end was sealed using Critoseal®Capillary Tube Sealant (Krackeler Scientific Inc, Albany, NY). After the initial stop, only the capillary tubes were allowed to fill prior to stopping. The tubing connected to the microchannel had continuous flow, as the feed loop was continued throughout to prevent thrombus formation. Capillary tubes from the parent branch were separated from those taken from the daughter branch, and all were centrifuged. After centrifugation, hematocrit was calculated by dividing the height of RBC column by the total height of the contents of the capillary tube and then multiplying by 100 to obtain percentage. These steps were repeated for RBC suspensions without DRP initially (N=91), followed by those with DRP at concentrations of 10 ppm (N=17), 5 ppm (N = 85), and 1 ppm (N=29). All hematocrit values were recorded and analyzed. All experiments were conducted at room temperature.

### Rat cremaster microcirculation

#### Animal preparation

All experimental protocols were approved by the Institutional Animal Care and Use Committee of University of Pittsburgh. Young Wistar male rats (N = 11) weighing 147 ± 14 g were anesthetized with isoflurane (3%) and maintained at 1–2% isoflurane. A 24G catheter was placed in the carotid artery and connected to a fluid filled catheter. Systemic blood pressure was recorded continuously using a Ponemah data acquisition system (DSI, St. Paul, MN). The right external jugular vein was cannulated with a 24G angiocatheter for DRP or saline infusion. An abdominal midline incision was made, the distal abdominal aorta was isolated and encircled with a 1 mm flow probe (Transonic, Inc, Ithaca, NY), and the incision was closed. The cremaster muscle was prepared for intravital microscopy as previously described [17]. Briefly, an incision was made in the left scrotal sac, the cremaster muscle was exposed, mounted and secured on a custom-designed microscope stage, and continuously irrigated with air-equilibrated 34°C HEPES buffer solution. Body temperature was maintained at 37°C by a heating pad.

#### Intravital Microscopy

Following surgery, the completed preparation was transferred to the stage of an intravital inverted microscope (Olympus BX51, Olympus, America, Inc., Center Valley, PA) linked to an ORCA-ER CCD camera (Hamamatsu, Bridgewater, NJ) recording at 8.3 frames per second. Recordings were made using the software package HCImage (Hamamatsu, Bridgewater, NJ). Microvessels were observed using bright-field (Olympus, Center Valley, PA, 100W Hg lamp) microscopy (condenser: Olympus WI-OBCD, numerical aperture (NA): 0.8) for the examination of CFL. A blue filter (435 nm, Howard Glass, Worcester, MA) was used to enhance the contrast between the RBCs and the background field. Cremaster muscle arterioles were examined with a 60× objective lens (Olympus; LUMPLFL 60XW 0.9 NA, WD 2 mm).

#### Experimental protocol

After being placed on the microscope stage, the preparation was allowed to equilibrate for 30 min, during which time the arteriolar network was scanned to find suitable arterioles with a diameter of 30 µm. In each animal one vessel was selected and recordings were made at baseline and 5 minutes after completion of DRP infusion and repeated every 10 minutes for 30 minutes. DRP solution (N = 5) was infused over 6 minutes (about 0.15–0.3 ml) to a final blood concentration of 1 ppm, similar to what was used in our previous study [2]. In an additional 6 rats, an equivalent volume of saline was infused at the same rate as the DRP, as control.

#### Data analysis

**Figure 2:**
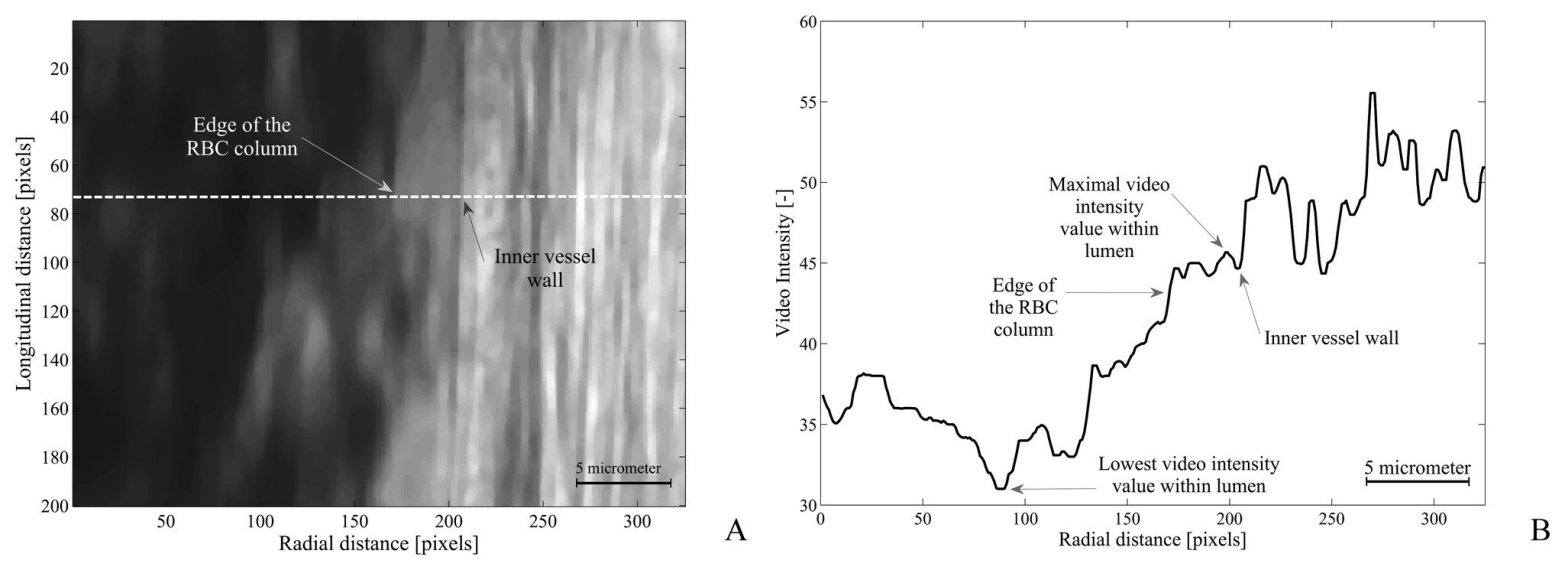
An arteriole with the video intensity profile corresponding to a single horizontal line of pixels A. Portion of a 43.9 µm arteriole used in the analysis. RBCs, appearing dark, are located on the left side of this panel. The vessel wall and muscle tissue are located on the right side of the image. B. The video intensity profile which corresponds to a single line of pixels indicated by the dotted line in the left panel. All 200 lines of the image were analyzed based on their video-intensity profile. The inner vessel wall, RBC column edge, and maximal and minimal pixel value within the vascular lumen are indicated by arrows.

All digital image processing procedures were performed with a commercially available image processing software package (MATLAB, Mathworks, Natick, MA, USA). For each recording, 4 frames were selected. To correct for movement of the tissue, the images were aligned using subpixel image registration by cross-correlation [18]. The 4 images per observation were analyzed sequentially. The applied model to measure CFL was adapted from Kim and coworkers [19,20]. First, a straight part of the vessel was selected as the region of interest (ROI). Next, the images were filtered with a median filter to remove binary noise. For each horizontal line of pixels (up to 200 lines, 21.5 µm length of vessel, per image), an intensity profile (VI) was created (see Figure 2B). By selecting a straight part of the vessel, the analysis lines were perpendicular to the vessel axis. The CFL was defined as the distance between the edge of the RBC column and inner vessel wall. The outer edge of the RBC column was arbitrarily set at 75% of the difference in pixel intensity between the maximum and minimum intensity on the luminal side of the vessel wall (Figure 2B), i.e. the difference between the darkest value within the RBC column and the brightest pixel value in the plasma layer. The inner vessel wall was defined as the dark line (Figure 2A), a marker for the luminal side of the endothelial cells [21-23] and is seen as the trough of the VI curve (Figure 2B). For the first line of the first frame the starting point of the algorithm (between RBC column and endothelium) was selected manually based on the image as can be seen in Figure 2A. For all subsequent lines, the point from where the algorithm starts was based on the location of the inner vessel wall and edge of the RBC column of previous lines. When the location of the vessel wall detected by the algorithm changed by more than 10 pixels between successive profiles, the algorithm stopped and continued with the next frame. CFL width was averaged over the length of the vessel in 4 different frames, resulting in a spatial and temporal averaged CFL width per measurement.

#### Statistical analysis

Significance of the effect of DRP on hematocrit in the *in vitro* setup was determined using an unpaired t-test. *In vivo*, effects of DRP on CFL, blood pressure, flow and vessel diameter within a group were analyzed using a Kruskal-Wallis test followed by Dunn’s multiple comparison post-hoc test. The effect of DRP on CFL comparing control with treated animals was assessed using an unpaired t-test. Results were considered statistically significant with P<0.05. Summary data are reported as means ± SEM, unless otherwise indicated.

## Results

### Microchannel bifurcation study

**Figure 3:**
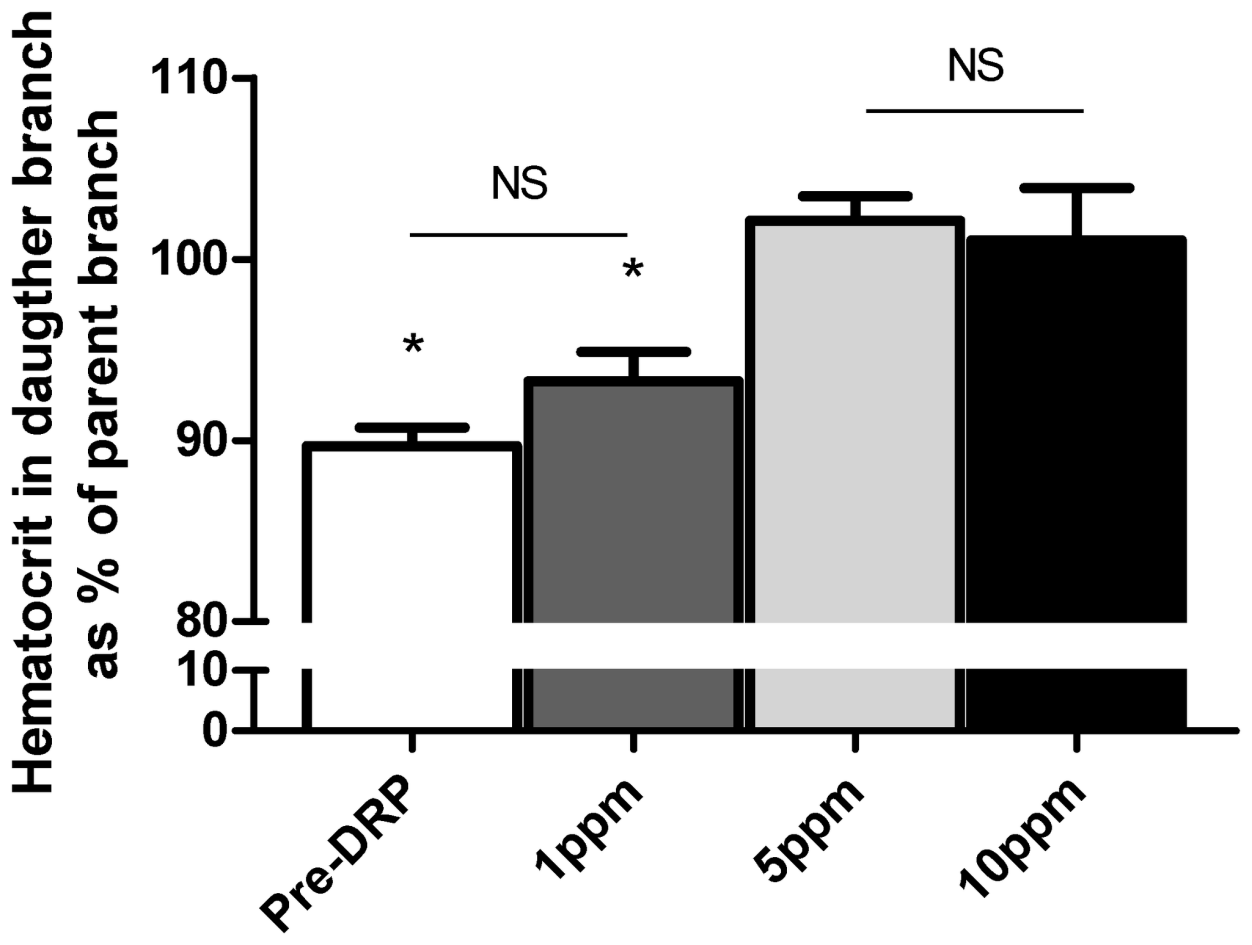
Hematocrit measured in daughter branch as percentage of parent branch In the control experiments and after addition of DRP at a concentration of 1 ppm the hematocrit in the daughter branch was still significantly less than in the parent branch. After increasing DRP concentration to 5 ppm and 10 ppm the hematocrit in the daughter branch was no longer different from the parent branch, thus demonstrating elimination of plasma skimming into the daughter branch. Values are mean ± SEM.

In the control experiment the hematocrit measured in the daughter branch was 89.7 ± 1.0% of the hematocrit in the parent branch (P<0.001). After administration of 1 ppm of DRP there was a trend towards an increase in hematocrit in the daughter branch (P=0.08) compared to the control experiment (see Figure 3), but the hematocrit of the daughter branch remained significantly less than that of the parent branch (93.3 ± 1.6% compared to parent branch, P<0.05). When the concentration of DRP was increased to 5 ppm, the hematocrit in the daughter branch was 102.1 ± 1.4% of the parent branch, and there was no longer any significant difference between daughter and parent branch hematocrit. A further increase in the DRP concentration to 10 ppm resulted in no significant difference between parent and daughter branch hematocrit, similar to that seen at a concentration of 5 ppm.

### Rat cremaster microcirculation

Baseline measurements of carotid pressure and aortic flow were 90.1 ± 3.9 mmHg and 11.3 ± 1.0 ml/min. Following DRP infusion, there was no statistically significant difference in these parameters at any of the time points up to 35 minutes after DRP infusion. Similarly these values were unchanged after saline infusion. Hematocrit taken during surgery (baseline: 30.8 ± 1.8% and 34.3 ± 1.5% for control and DRP group, respectively) and after completion of the experiment (25.8 ± 2.9% for control and 30.9 ± 1.9% for DRP, P<0.05 compared to baseline) was not statistically different comparing the control group with the animals treated with DRP. Vessel diameter was 32.6 ± 1.7 µm (range 25–44 µm) at baseline and did not change during the experiment for the animals treated with DRP or saline.

**Figure 4:**
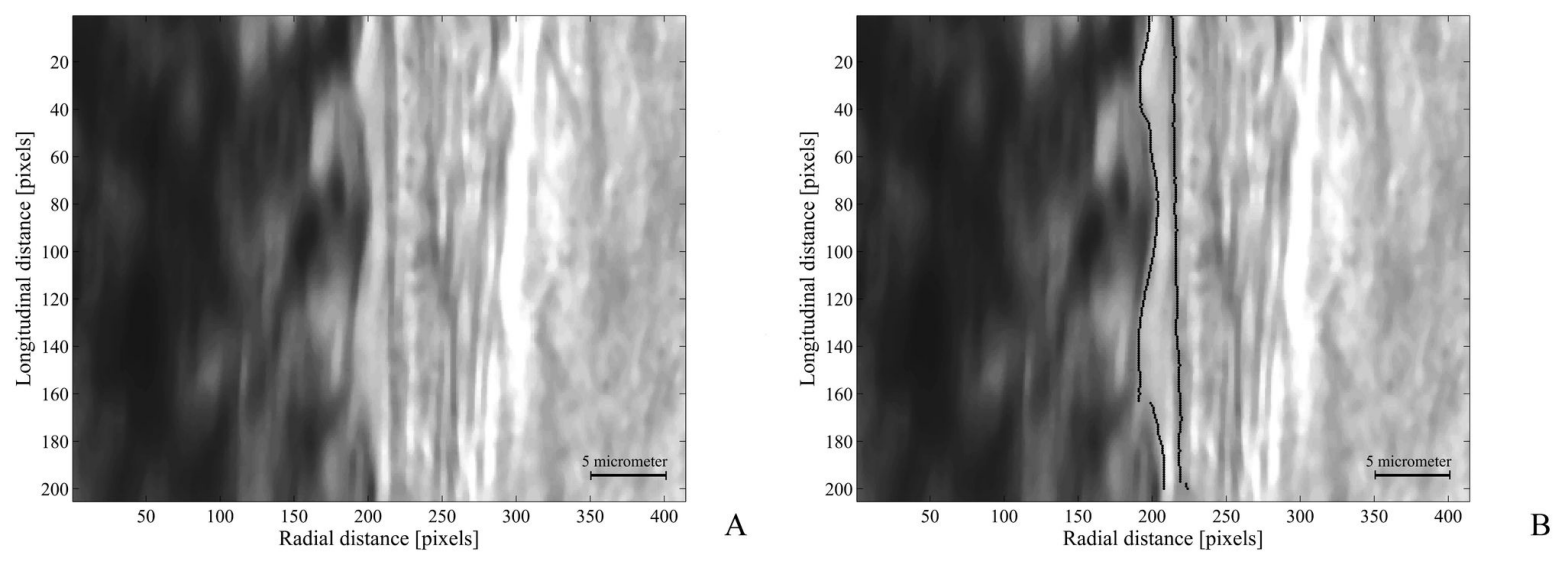
Data analysis An example of a section of an artery before data analysis (Figure 4A) and after tracing the RBC column and vessel wall over a length of 200 pixels (Figure 4B). The difference between vessel wall and RBC column is defined as the CFL.

An example of a frame before and after analysis of the intensity profiles is given in Figure 4. In Figure 4A the original image is shown, with the RBC column on the left, and the vessel wall and surrounding tissue on the right. In the image on the right (Figure 4B) the same arterial segment is depicted, but now with tracing (black lines) of the edge of the RBC column and the luminal surface of the vessel wall using the automated algorithm presented in the methods section.

**Figure 5:**
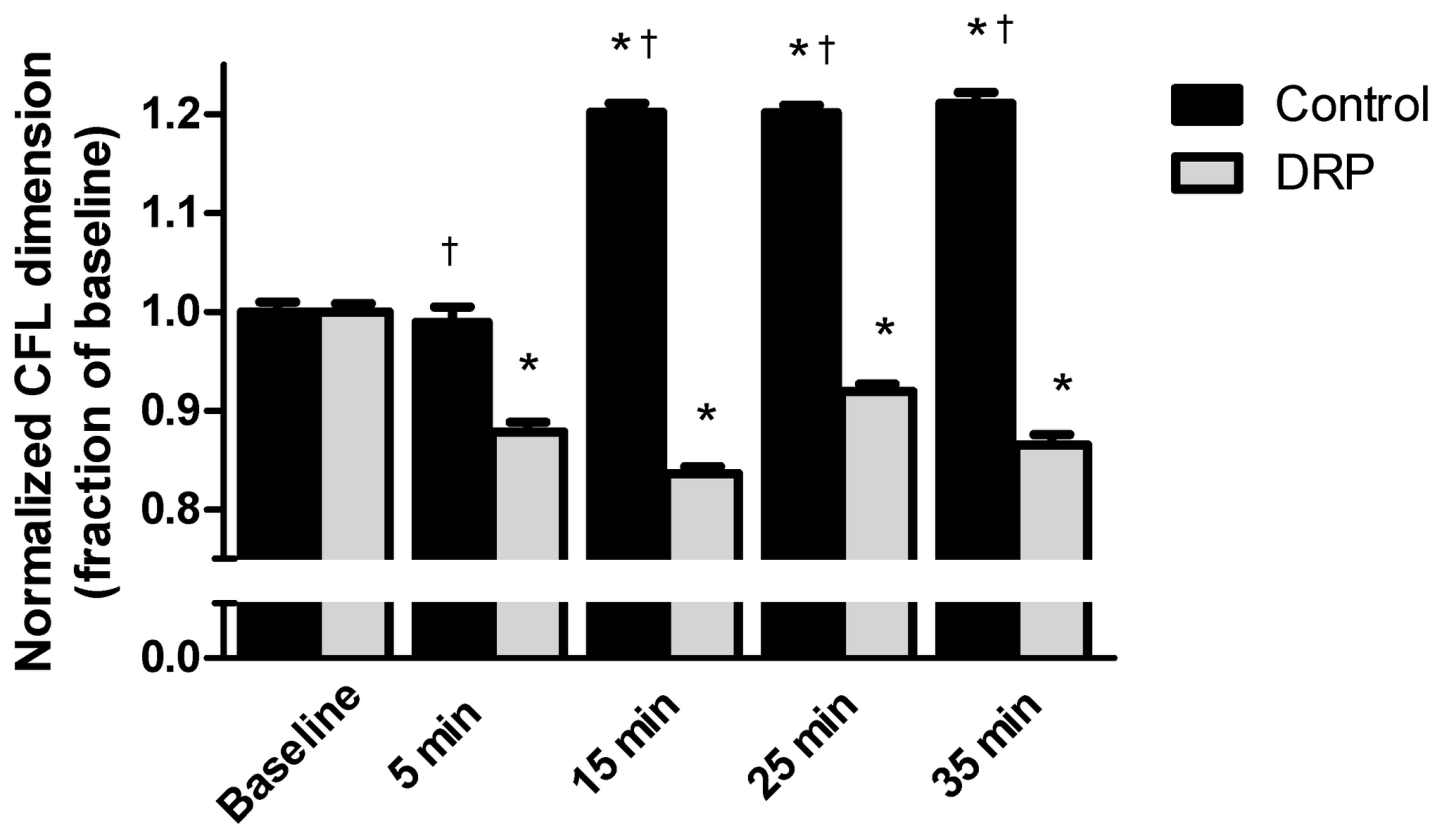
Cell free layer width Cell-free region (CFL) normalized to baseline for control (black, N=6 animals) and DRP treated animals (gray, N=5 animals) presented as mean ± SEM. In the control group there is a significant increase in CFL at 15, 25 and 35 minutes of 20.2 ± 0.02% (SEM), 20.1 ± 0.01% and 21.1 ± 0.01%, respectively. After administration of DRP there is a significant reduction in CFL compared to baseline, at 5 min CFL is 87.8 ± 0.02% of baseline and 83.6 ± 0.01%, 92.0 ± 0.01%, and 86.5 ± 0.01% at 15, 25 and 35 minutes, respectively. * P<0.05 from corresponding baseline dimensions before administration of DRP or saline, † P<0.05 compared to DRP at same moment in time.

The CFL data was normalized to baseline. Comparing the normalized CFL data, a significant difference was found comparing the control animals to those treated with DRP (P<0.001) for each time point after baseline. The CFL observed in the control animals was significantly larger compared to those animals treated with DRP. When comparing baseline to the different time points, the CFL was significantly reduced in the animals treated with DRP. In the control animals the CFL was significantly increased compared to baseline at 15, 25 and 35 minutes after the infusion of saline (P<0.05), except at the 5 minute time-point (Figure 5).

## Discussion

In this study we have shown that DRP eliminates plasma skimming in a 50 µm T-bifurcation model, consisting of cylindrical microchannels. This effect aligns with previous observations showing a reduction in CFL width in square microchannels using flow visualization techniques [8,12]. The use of cylindrical channels, which were produced using a suspended wire technique, is arguably more relevant, given that their geometry is more similar to microvessels in vivo. In these studies we observed an increase in hematocrit in the daughter branch, after addition of DRP to RBC suspension. More importantly, in this work we demonstrated that administration of DRP at a concentration of as little as 1 ppm reduced CFL significantly compared to baseline and the control group, in the rat cremaster microcirculation (Figure 5). These data demonstrate, for the first time, that the *in vitro* observations showing a decrease in CFL with DRP do occur *in vivo*. Based on our *in vitro* and *in vivo* observations, this suggests that DRP reduces plasma skimming, increases arteriolar side branch hematocrit, thereby diverting more RBCs into the capillary bed. This redistribution of RBCs due to a reduction in plasma layer width within the microvascular network [24] may explain the previously observed DRP mediated enhancement of capillary perfusion.

### Red blood cell redistribution after DRP infusion

Infusion of DRP has been associated with increased survival after hemorrhagic shock [3,6,8], improved perfusion after myocardial infarction [5,25] or in the presence of a coronary artery stenosis [1,26], and reduced risk for atherosclerosis in areas with low shear stress [27,28]. The mechanism of how DRP influences perfusion is, however, not completely understood. Recently, a redistribution in RBCs (e.g., an increase in the number of RBCs in a branch or reduction in plasma skimming) was observed [12]. The authors used an *in vitro* model consisting of rectangular channels, however, the vasculature consists of round-shaped vessels. When implementing the experiment in a model with round microchannels we observed a similar phenomenon, a redistribution of RBCs increasing the hematocrit in a side branch up to the level of the parent vessel. Importantly, this effect was DRP concentration dependent, at a concentration of 1 ppm there was a trend towards an increase in hematocrit in the side branch and at 5 and 10 ppm there was no longer a difference between hematocrit in the main channel and a side branch.

In these studies a hematocrit of 30% was chosen since *in vivo* microcirculatory hematocrit are found to be significantly smaller than the systemic hematocrit [29-31] due to the Fåhraeus phenomenon. In fact, the hematocrit used in our *in vitro* experiments was equal to the hematocrit found in arterioles with a comparable diameter (50 µm) in the rat cremaster [32]. Similar to the *in vitro* studies using rectangular microchannels [12,33] we used bovine blood. By using the same type of blood, and therefore minimizing changes related to rheology, we could more thoroughly investigate difference between microchannel geometry (square versus round microchannels).

### Reduced cell-free layer after DRP infusion

So far, the effect of DRP on perfusion has been studied using *in vitro* microchannel models. However, microvessels are distinct from microchannels in that they are elastic, lined with endothelium, relatively short, and may be irregular in shape. Furthermore, CFL formation in arteriolar bifurcations has different characteristics compared to CFL formation in microchannels. Ong and coworkers observed spatial variability of the CFL formation in the vicinity of an arteriolar bifurcation due to blood flow separation at the bifurcation, leading to heterogeneities of the layer formation not only within individual vessel segments but also between downstream vessels at the bifurcation [34,35]. Data from an *in vitro* setup, therefore, cannot be completely extrapolated to an *in vivo* setting. To further elucidate potential mechanisms behind the DRP effects on the microcirculation we performed experiments in the rat cremaster microvessels *in vivo* where one group was treated with saline and the other group with DRP (1 ppm). Importantly, the CFL was measured at a distance exceeding two vessel diameters away from any bifurcation, to minimize any potential confounding effects of the bifurcation on the CFL [34].

We used the same concentration of DRP that successfully enhanced perfusion in the canine myocardium seen in our previous study [1,2]. Adding DRP to a final blood concentration of 1 ppm resulted in a significant decrease in CFL compared to baseline, and to control animals. In the absence of DRP the CFL increased in time. The increase in CFL, as observed in our control experiments, might have been caused by a change in plasma protein composition as has been seen to occur in acute inflammation (so-called acute phase reactants), increasing RBC aggregation [36]. We did observe a small number of adhering white blood cells to the endothelium in both the control as well as DRP treated animals during the experiments. Adhesion of the white blood cells itself might also have contributed to the increased CFL as was shown using a two-dimensional computational model [37]. The effect of RBC aggregation on CFL might, however, have been limited due to high shear conditions at normal perfusion pressures/flows [38]. Another possible mechanism that could have contributed to the increase in CFL is a reduction in hematocrit. In this invasive procedure, there was a 4.9 ± 1.7% drop in hematocrit over the course of the study (P<0.05) which could explain the increase in CFL width. However, this same percentage reduction in hematocrit was observed in the treatment group as well.

### Microvascular perfusion

The width of the CFL is influenced by many parameters, such as hematocrit, presence of endothelial glycocalyx, vessel diameter, RBC aggregation and deformability, and mean flow velocity [39]. In our study there was neither a difference in hematocrit between the DRP treated animals and control group, nor a change in vessel diameter or aortic blood flow. The change in CFL can, therefore, not be explained by a change in hemodynamic parameters and seems to be due to administration of DRP. Based on our *in vitro* and *in vivo* observations, we hypothesize that a reduced CFL results in less plasma skimming and a more homogeneous flow and hematocrit distribution [24] throughout the vascular tree. Under normal conditions, a disparity has been seen in different vessel segments of a capillary network all fed by the same terminal arteriole, the network Fåhraeus effect [40]. The entire network Fåhraeus effect is generated by a positive correlation of flow rates and hematocrit in segments of a microvascular network. Unequal partition of hematocrit at successive arteriolar branch points leads to a high degree of heterogeneity in the hematocrit of individual segments. Furthermore, the tendency of RBCs to follow the higher-flow pathway at each bifurcation results in a strong correlation between hematocrit and flow velocity [36,40]. DRP induced reduction in plasma skimming could reduce the uneven separation of RBC and plasma at the terminal arteriole-capillary bifurcations, resulting in a more homogeneous microvascular perfusion and increased capillary hematocrit [41,42]. Furthermore, a reduction in CFL is expected to result in an increase in apparent, or effective viscosity [43], which in turn would increase wall shear stress, and therefore vascular resistance. However, this would be counteracted, at least in part, by the release of endothelial NO which would result in vasodilation [44-46]. The magnitude of this shear mediated vasodilation is known to be heterogeneous within the arteriolar compartment [44,47]. Nevertheless, in a previous study we have shown that intravenous DRP administration reduced the pressure loss between the aorta and the arteriolar compartment (arteriolar pressure increased by 22%), suggesting a significant decrease in hydraulic resistance between the aorta and arteriolar compartment [48]. Therefore, DRP administration has a beneficial effect on flow, through a net reduction in vascular resistance, which appears to arise, in part, from the diminished thickness of the cell-free layer and attendant increases in wall shear stress.

### Data analysis

Analysis of CFL was based on the technique developed by Kim et al. [19,20]. In their study the CFL was measured using a high speed camera and CFL was measured in time at a single line. Their results indicated that CFL is not constant for a given location. In our study we adapted their method and measured CFL in time and along the length of a segment of an artery, without the use of a high speed camera. The video intensity profile for up to 200 lines in 4 different frames of the recording was analyzed. In each frame a minimal of 56 lines could be analyzed, and when comparing the animals treated with DRP with those animals receiving saline only the first 56 lines of each frame were taken into account, resulting in 224 measurements of the CFL per point in time per animal. On average 184 lines could be analyzed per frame. The inner vessel wall was defined as the dark line, a marker for the endothelium [21-23] and could be found as a trough in the VI curve (Figure 2B). Due to minimal data filtering, noise could be seen in our intensity curves (Figure 2B). Consequently, the maximal light intensity gradient, as was reported in literature [20], resulted in inaccurate tracing of the RBC column. We had to redefine the edge of the RBC column and used 75% of the maximal VI difference that could be found in the lumen of the vessel (Figure 2B). The highest value was found near the endothelium in the plasma layer and the lowest VI value in the (center of the) RBC column. Detection of the edge of the RBC column was visually validated. The results obtained with our method appeared to be in good agreement with visually detecting the edge of the RBC column (Figure 4B). After spatial and temporal averaging of the data a mean CFL of 2.93 ± 0.02 µm (range 2.14-3.75 µm) was measured at baseline, pooled data of DRP and control group. A value well within the range reported in literature using similar detection methods with different definitions of detecting the RBC column [19,20] in vessels with diameters corresponding to those included in our study.

### Study limitations

Unlike the *in vitro* setup, a significant effect of DRP on CFL was observed at a concentration of 1 ppm in the rat cremaster tissue. Since it was proven that the change in CFL occurs as a result of DRP administration, and the effect was obtained at 1 ppm, it was decided to not repeat the experiments at higher DRP concentrations. However, studies to demonstrate a dose response effect *in vivo* would be of value. Based on preliminary data on the effect of DRP concentration (1-5 ppm) on RBC velocity, aortic blood pressure and blood flow, a further change in CFL with increasing concentration of DRP might be expected. Furthermore, in the *in vitro* studies we found a dose dependent effect of DRP on the magnitude of plasma skimming, which suggests that there is a dose dependent effect on the CFL.

We used a camera with a frame rate of 8.3 frames per second. Mean centerline RBC velocity was between 4-5.5 mm/sec and although velocity of the RBCs was less near the vessel wall, smearing of the RBCs has been observed. Consequently, some RBCs look elongated since they moved during recording of the frame. However, the low frame rate worked as a low pass filter, resulting in a better averaged CFL width estimation due to a temporal averaging within a single frame. The spatial resolution of the images was 9.30 pixels per micrometer. The minimum difference in CFL that could be detected is, therefore, 0.11µm. The change in CFL observed in the current study was 0.5µm and in different direction for control (increase in CFL) as for DRP treated animals (reduction in CFL). Thus, the spatial resolution formed no limitation for detecting changes in CFL width.

When analyzing the data, a part of the vessel was selected that was relatively straight. Small irregularities in the shape of the vessel were not corrected for. Therefore, it is possible that not all measurements of the distance between the inner vessel wall and the RBC column were exactly perpendicular to the vessel wall. However, when comparing our measurements we did use the same part of the vessel for all 5 stages in our experiment, therefore any error introduced by irregularities in vessel wall will have no effect on the outcome of our study. The absolute CFL dimension might have been influenced by not taking irregularities in vessel wall into account but, as mentioned in the discussion, our data is well within the range reported by others [19,20].

Last, the hematocrit values measured in our study in both the control group and DRP treated animals are lower as those values reported in literature for rats (35-45% hematocrit [35,38]). During surgery, blood loss was observed and fluids were given to compensate for this loss of volume (up to 1 ml), causing a reduction in the hematocrit. As a result, an increase in absolute CFL width was observed in our experiments, for both groups. Nevertheless, the measured values of the CFL are well within range of reported values for vessels with corresponding diameter.

## Conclusion

The findings of the current study demonstrated 1) reduction in plasma skimming by DRPs using round microchannels, and for the first time, 2) reduction in CFL in an *in vivo* animal model of the rat cremaster muscle by nanomolar concentrations of intravenous DRPs. Our *in vitro* and *in vivo* findings taken together, suggest that through a reduction in plasma skimming, DRPs cause redistribution of RBCs within the microvascular network, which would promote delivery of RBCs to the capillary bed, and may explain the previously observed DRP induced increase in capillary perfusion. These findings provide novel insight into the microvascular mechanisms of DRPs.
